# Electro-catalytic amplified sensor for determination of N-acetylcysteine in the presence of theophylline confirmed by experimental coupled theoretical investigation

**DOI:** 10.1038/s41598-020-79872-0

**Published:** 2021-01-13

**Authors:** Mohsen Keyvanfard, Hassan Karimi-Maleh, Fatemeh Karimi, Francis Opoku, Ephraim Muriithi Kiarii, Poomani Penny Govender, Mehdi Taghavi, Li Fu, Aysenur Aygun, Fatih Sen

**Affiliations:** 1 Department of Chemistry, Majlesi Branch, Islamic Azad University, Majlesi, Iran; 2 School of Resources and Environment, University of Electronic Science and Technology of China, Xiyuan Ave, Chengdu, 611731 People’s Republic of China; 3 Department of Chemical Engineering, Quchan University of Technology, Quchan, Iran; 4 Department of Chemistry, University of Johannesburg, P.O. Box 17011, Doornfontein Campus, Johannesburg, 2028 South Africa; 5 Polymer Chemistry Research Laboratory, Faculty of Science, Shahid Chamran University, 61357-43337 Ahvaz, Iran; 6 College of Materials and Environmental Engineering, Hangzhou Dianzi University, Hangzhou, 310018 China; 7 Sen Research Group, Biochemistry Department, Faculty of Arts and Science, Dumlupınar University, Evliya Çelebi Campus, 43100 Kütahya, Turkey

**Keywords:** Biochemistry, Biological techniques, Biotechnology, Chemistry, Engineering, Materials science, Nanoscience and technology

## Abstract

The 1,l/-bis(2-phenylethan-1-ol)ferrocene, 1-butyl-3-methylimidazolium hexafluoro phosphate (BMPF6) and NiO-SWCNTs were used to modify carbon paste electrode (BPOFc/BMPF6/NiO-SWCNTs/CPE), which could act as an electro-catalytic tool for the analysis of *N*-acetylcysteine in this work. The BPOFc/BMPF6/NiO-SWCNTs/CPE with high electrical conductivity showed two completely separate signals with oxidation potentials of 432 and 970 mV for the first time that is sufficient for the determination of *N*-acetylcysteine in the presence of theophylline. The BPOFc/BMPF6/NiO-SWCNTs/CPE showed linear dynamic ranges of 0.02–300.0 μM and 1.0–350.0 μM with the detection limit of ~ 8.0 nM and 0.6 μM for the measurement of *N*-acetylcysteine and theophylline, respectively. In the second part, understanding the nature of interaction, quantum conductance modulation, electronic properties, charge density, and adsorption behavior of *N*-acetylcysteine on NiO–SWCNTs surface from first-principle studies through the use of theoretical investigation is vital for designing high-performance sensor materials. The *N*-acetylcysteine molecule was chemisorbed on the NiO–SWCNTs surface by suitable adsorption energies (− 1.102 to − 5.042 eV) and reasonable charge transfer between *N*-acetylcysteine and NiO–SWCNTs.

## Introduction

The thiolic biological compounds such as cysteine, *N*-acetylcysteine, homocysteine, glutathione, captopril etc. play important roles in human health^1–4^. *N*-acetylcysteine as thiol drugs showed much application in the treatment of chest pain, Alzheimer disease, and overdose with acetaminophen^5–10^. In addition, *N*-acetylcysteine is used for the control of lipoprotein and homocysteine levels that are harmful to the human body at high levels^11^. On the other hand, the high consumption of *N*-acetylcysteine can increase risk of vomiting, nausea, and constipation which is very significant for the investigation of *N*-acetylcysteine in real samples^12–15^.

On the other hand, theophylline is a methylxanthine drug with a wide range of application for the treatment of severe asthma, prevention of contrast-induced nephropathy, chronic bronchitis, chest tightness, and wheezing. According to the scientific report, the combination of theophylline and *N*-acetylcysteine is useful for treating chronic obstructive pulmonary disease^16^. According to the report by Mirhosseini et al., the simultaneous use of *N*-acetylcysteine and theophylline can reduce the side effects in the treatment of stomach discomfort, cardiac complications, etc^16^. Therefore, theis research work focused on the simultaneous investigation of *N*-acetylcysteine and theophylline in a nano-molar level using a selective electrochemical tool.

Due to their selective response and low-cost, the electrochemical sensors are a better selection compared to other analytical methods for the determination of drugs^17–20^. High overvoltage of *N*-acetylcysteine and theophylline at the bare electrode surface is a major issue for the analysis of this drug at low levels^7,12,21^. Therefore, modification of electrodes is vital for the investigation of *N*-acetylcysteine at low levels^22^. Recent studies have shown that nanomaterials with a wide range of applications and different properties^23–33^ along with room temperature ionic liquids can enhance the sensitivity of electrodes for the determination of drugs and biological samples with a weak oxidation signal^34–36^. On the other hand, the simultaneous amplification of electrodes with nanomaterials and ionic liquids exhibited high performance for improving oxidation/ reduction signals^37–39^. In addition, electro-catalytic interaction between electroactive mediators with analytes is a good strategy for improving selectivity of electroanalytical sensors^40–46^.

In this research, modified electrochemical sensor (BPOFc/BMPF6/ NiO-SWCNTs/CPE in this case) was fabricated as a powerful tool for determination of *N*-acetylcysteine in drug samples. The BPOFc/BMPF6/NiO-SWCNTs/CPE displayed high sensitive (owing to the existence of ionic liquid and NiO-SWCNTs) capability for the investigation of *N*-acetylcysteine in the presence of theophylline for the first time. Moreover, the adsorption behavior and electronic properties of *N-*acetylcysteine with NiO–SWCNTs was investigated through a theoretical study. Four (4) adsorption sites were modeled and the lowest energy configurations were identified using density functional theory (DFT) calculations with the aim of illustrating the performance of adsorption sites and interfacial effects. A detailed analysis of charge density, the density of states (DOS) and charge transfer mechanism of *N-*acetylcysteine before and after adsorption onto a NiO–SWCNTs surface was also examined. The DFT method has shown more understanding of the adsorption reaction and nature of interactions of the target molecules with the base material, as well as, changes in the electronic properties^47^. The theoretical investigations confirm that the presence of NiO/SWCNTs can increase the electrical conductivity of the carbon paste medium and help the adsorption of *N-*acetylcysteine at the surface of the proposed sensor for better catalytic interaction with BMPF6.

## Experiments

### Chemicals and instrument

*N*-acetylcysteine, nickel nitrate, theophylline, and BMPF6 were bought from Sigma-Aldrich. Single wall carbon nanotubes functionalized with COOH was purchased from Neutrino Company, Iran. The BPOFc and NiO/SWCNTs were synthesized according to papers reported by Karimi-Maleh et al.^48,49^. An Autolab PGSTAT 12, potentiostat/galvanostat system with NOVA software was used for recording all of the voltammetric signals. The Zeiss-EM10C-100 kV and X’ Pert Pro instruments were used for TEM and XRD investigation, respectively. MAP analysis of nanocomposite was recorded by a FESEM instrument model MIRA3TESCAN-XMU with Page and linear analysis software.

### Fabrication of BPOFc/BMPF6/NiO-SWCNTs/CPE

0.01 g BPOFc + 0.05 g NiO-SWCNTs + 0.94 g graphite was dispersed in diethyl ether. At room temperature, the solvent evaporated and then paraffin oil and BMPF6 oils were used for the preparation of paste. The BPOFc/BMPF6/NiO-SWCNTs/CPE paste was inserted in a glass tube with copper wire as a conductor of electricity.

### Real sample preparation

The water and pharmaceutical serum samples were purchase from the local market and pharmacy and directly used for electrochemical analysis. Tablet samples were purchased from local pharmacy and then were completely ground and homogenized. Next, their calculated values were weighed and then dissolved in 50 mL of water/ethanol solution and the mixture was filtered for real sample analysis.

### Computational details

The electronic and structural properties of *N-*acetylcysteine adsorbed onto a NiO–SWCNTs surface was investigated using the plane-wave DFT calculations as implemented in the Cambridge Serial Total Energy Package code^50^. The generalized gradient approximation (GGA) of the Perdew–Burke–Ernzerhof (PBE) functional^51^ and ultrasoft pseudopotentials^52^ were used to describe the exchange–correlation and core-valence electron. The adsorption energies were calculated using the dispersion correction by Grimme^53^, since van der Waals interactions were anticipated to affect the adsorption energies. A vacuum gap of 20 Å was used to prevent the interactions between the periodic slabs perpendicular to the surface, resulting in a simulation supercell of 10.393 × 8.520 × 35.073 Å^3^. The Monkhorst–Pack^54^ with *k*-mesh of 4 × 1 × 1 was used to sample the Brillouin zone. The wave functions of the valence electron were described using a plane-wave basis set with a cut-off energy of 400 eV. To account for the metallic behavior of NiO_2_ (the oxidation states of each element in NiO_2_ are + 4 (Ni) and –2 (O)), the atomic positions were optimized via the Broyde*N-*Fletcher–Goldfarb–Shanno scheme^55^ with an energy convergence criterion, force, and displacement of less than 10^−6^ eV/atom, 0.3 eV/Å, and 0.01 Å, respectively. However, all other atoms and lattice vectors on the top layer of the slab were allowed to relax, since surface adsorption occurred on the topmost layer. The Hirshfeld’s analysis^56^ was used to evaluate the charge transfer between *N-*acetylcysteine and NiO–SWCNTs.

The stability of *N-*acetylcysteine adsorption on the NiO–SWCNTs surface was evaluated by calculating the adsorption energy (*E*_ads_):

1 $$ E_{ads} = E_{{N{-}acetylcysteine@NiO{-}SWCNTs}} - E_{{N{-}acetylcysteine}} - E_{NiO} - E_{SWCNTs} , $$

where $$E_{{N{-}acetylcysteine@NiO{-}SWCNTs}}$$, $$E_{{N{-}acetylcysteine@NiO{-}SWCNTs}} ,$$ $$E_{{N{-}acetylcysteine}} ,$$ $$E_{NiO} $$ and $$E_{SWCNTs}$$ are the sum of the energies of *N-*acetylcysteine which are adsorbed on NiO–SWCNTs surface, monolayers of *N-*acetylcysteine, NiO and SWCNTs, respectively. Generally, a negative *E*_ads_ signifies that the adsorption process was exothermic and energetically stable^57^.

The highest occupied molecular orbital (HOMO) − lowest unoccupied molecular orbital (LUMO) gap (HLG) was evaluated following Eq. (2):

2 $$ E_{g} = E_{LUMO} {-}E_{HOMO} $$

where $E_{LUMO}$ and $E_{HOMO}$ are the energies of the LUMO and HOMO, respectively. The electronic sensitivity of the NiO–SWCNTs towards the adsorption of *N-*acetylcysteine was assessed by calculating the change in the HLG^58^:

3 $$ {\Delta }E_{{\text{g}}} = \left[ {\left( {E_{{{\text{g}}2}} - E_{{{\text{g}}1}} } \right)/E_{{{\text{g}}1}} } \right] \times 100 $$

where $E_{{{\text{g}}1}}$ and $E_{{{\text{g}}2}}$ represent the HLG before and after adsorption.

## Results and discussion

### NiO/SWCNTs characterization

**Figure 1:**
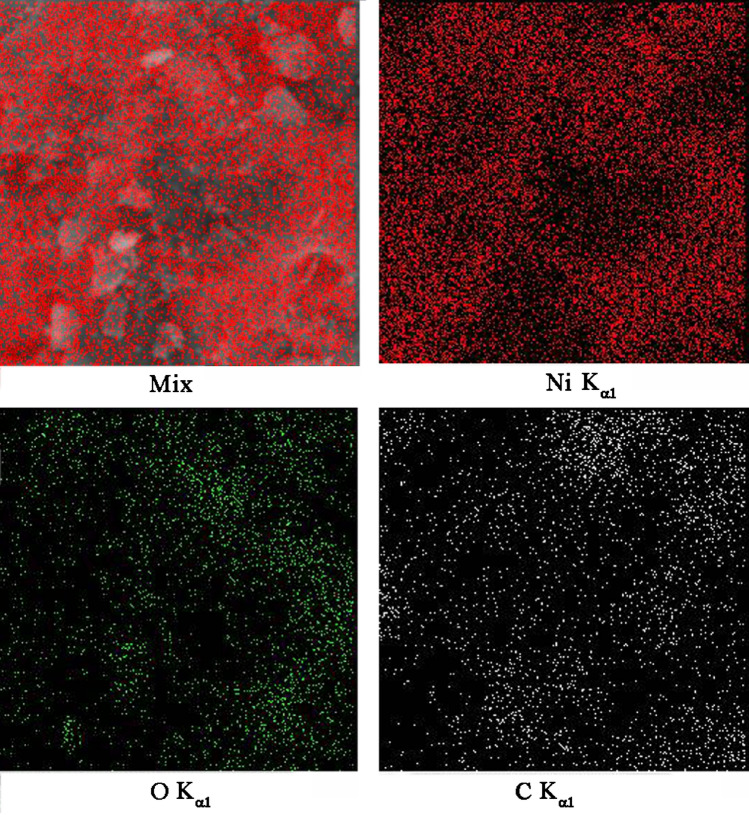
(**A**) MAP analysis image of NiO/SWCNTs nanocomposite.

**Figure 2:**
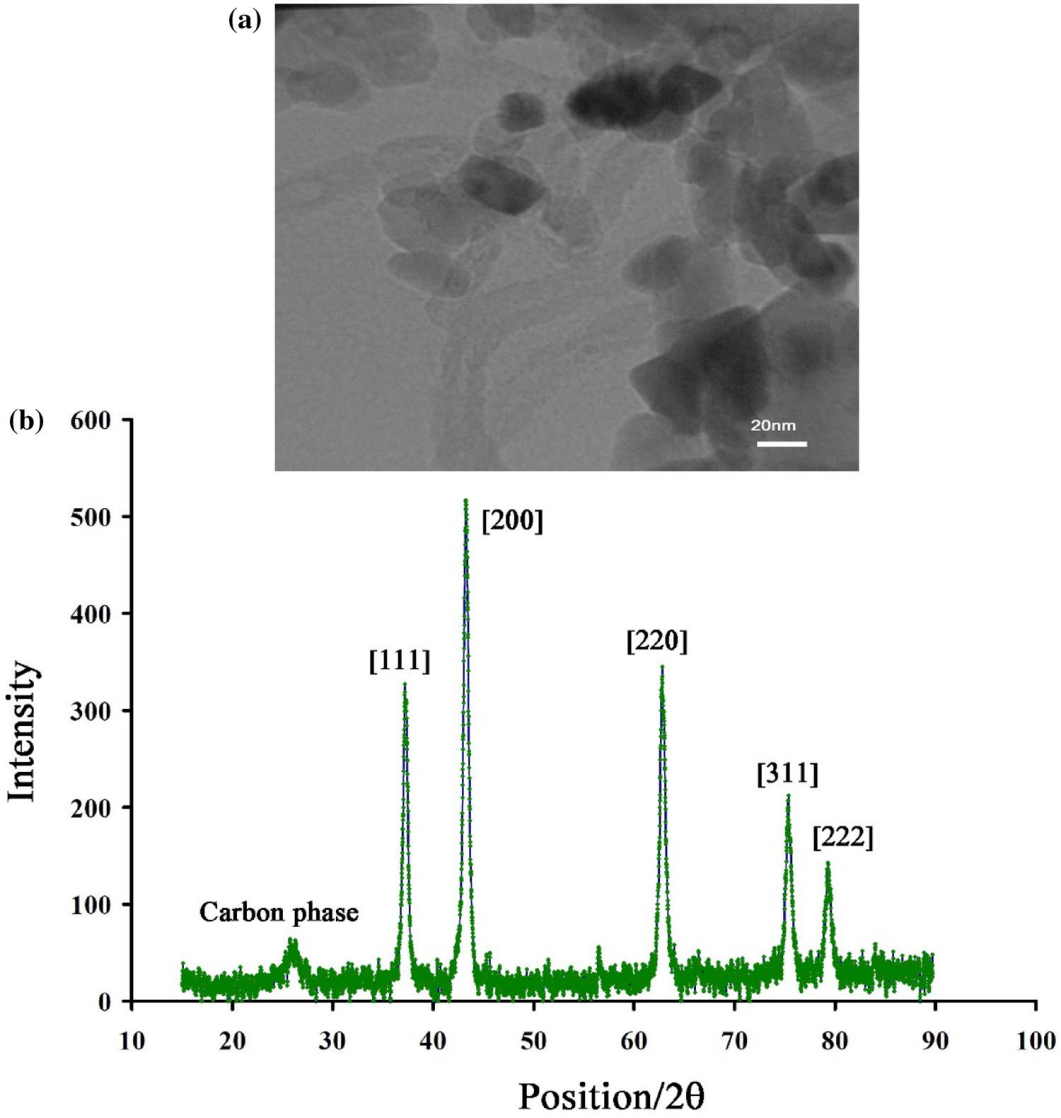
(**a**) TEM image of NiO/SWCNTs nanocomposite. (**b**) XRD pattern of NiO/SWCNTs nanocomposite.

The elemental analysis of NiO-SWCNTs is shown in Fig. 1. The existence of C, Ni and O elements confirm the purity of synthesized NiO-SWCNTs nano-composites by the recommended procedure. The decoration of NiO/NPs at functional SWCNTs was characterized by TEM method (Fig. 2A). The presence of nickel oxide nanoparticles on the single-wall carbon nanotubes surface is well represented in Fig. 2A. In contrast, the XRD patterns of NiO-SWCNTs confirm the occurrence of (002) at 2θ ~ 26° plane relative to carbon nanotubes and other planes, i.e. (111), (200), (220), (311) and (222) at positions of 37.171°, 43.231°, 62.791°, 75.321° and 79.191° relative to NiO nanoparticle with FCC structure (Fig. 2).

### Electro-catalytic determination of N-acetylcysteine by BPOFc/BMPF6/NiO-SWCNTs/CPE

The electro-oxidation of *N*-acetylcysteine with thiolic structure is relative to pH of solution. The electro-catalytic interaction between BPOFc and *N*-acetylcysteine was optimized by recording signals of 1.0 mM drug at BPOFc/BMPF6/NiO-SWCNTs/CPE surface with pH ranges of 4.0–8.0. According to obtained data (not shown), it is observed that maximum electro-catalytic interaction could occur at pH = 7.0 and this pH was chosen as the best condition.

**Figure 3:**
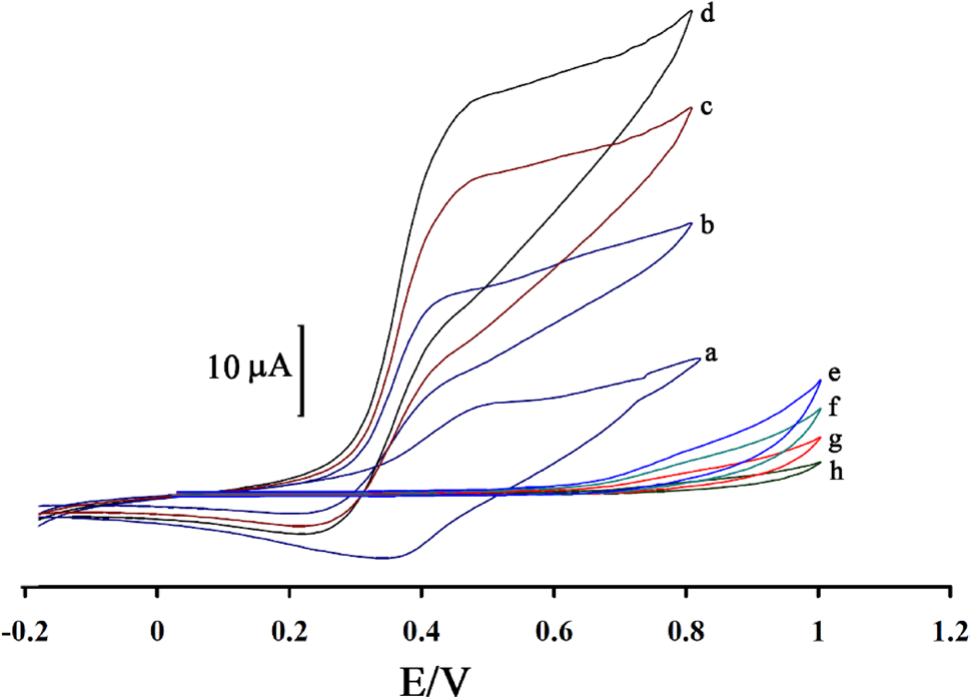
Cyclic voltammograms of BPOFc/BMPF6/NiO-SWCNTs/CPE (**a**); BPOFc/NiO-SWCNTs/CPE in the presence of 1.0 mM N-acetylcysteine (**b**); BPOFc/BMPF6/CPE in the presence of 1.0 mM N-acetylcysteine (**c**); BPOFc/BMPF6/NiO-SWCNTs/CPE in the presence of 1.0 mM N-acetylcysteine (**d**); BMPF6/SWCNTs/CPE in the presence of 1.0 mM N-acetylcysteine (**e**); BMPF6/CPE in the presence of 1.0 mM N-acetylcysteine (**f**); SWCNTs/CPE in the presence of 1.0 mM N-acetylcysteine (**g**) and CPE (**h**) in the presence of 1.0 mM N-acetylcysteine. Condition; pH = 7.0 and scan rate 20 mV/s.

The signal for the oxidation of 1.0 mM N-acetylcysteine was recorded at BPOFc/NiO-SWCNTs/CPE (Fig. 3, curve b), BPOFc/BMPF6/CPE (Fig. 3, curve c), BPOFc/BMPF6/NiO-SWCNTs/ CPE (Fig. 3, curve d), BMPF6/NiO-SWCNTs/CPE (Fig. 3, curve e), BMPF6/CPE (Fig. 3, curve f), NiO-SWCNTs/CPE (Fig. 3 curve g) and CPE (Fig. 3, curve h). On the other hand, BPOFc/BMPF6/NiO-SWCNTs/CPE exhibited an oxidation/reduction signal with ∆Ep = 130 mV that confirms quasi-reversible behavior of BPOFc/BMPF6/NiO-SWCNTs/CPE in the aqueous solution (curve a). The increasing oxidation signal of BPOFc/BMPF6/NiO-SWCNTs/CPE and simultaneous decrease in reduction signal of mediator after addition of 1.0 mM N-acetylcysteine, confirms an EC^/^ interaction^59,60^ between mediator and N-acetylcysteine on the surface of BPOFc/BMPF6/NiO-SWCNTs/CPE. The comparison of the electro-catalytic oxidation signal of *N*-acetylcysteine at the surface of BPOFc/BMPF6/NiO-SWCNTs/CPE with its signal at BPOFc/NiO-SWCNTs/CPE and BPOFc/BMPF6/CPE confirmed that the conductivity of electrode surface could be enhanced by the existence of NiO-SWCNTs and BMPF6. This increase in conductivity helps to improve oxidation current and decrease oxidation potential. In addition, the comparison of the electro-catalytic oxidation signal of *N*-acetylcysteine at the surface of BPOFc/NiO-SWCNTs/CPE with its signal at BPOFc/BMPF6/CPE shows that reduction in oxidation potential at the surface of BPOFc/NiO-SWCNTs/CPE is more than its reduction at the surface of BPOFc/BMPF6/CPE. This point can be related to the high viscosity of BPOF, which makes it difficult to access the electrode surface. On the other hand, oxidation signal of *N*-acetylcysteine showed a weak signal at the surface of CPE. After modification of CPE with NiO-SWCNTs or BPOFc, the oxidation signal of *N*-acetylcysteine was improved, that could be related to high conductivity of mediators at the surface of CPE. In addition, after modification of CPE with NiO-SWCNTs and BPOFc, a better oxidation signal for *N*-acetylcysteine that is relative to synergic effect of the two mediators at surface of CPE is observed.

**Figure 4:**
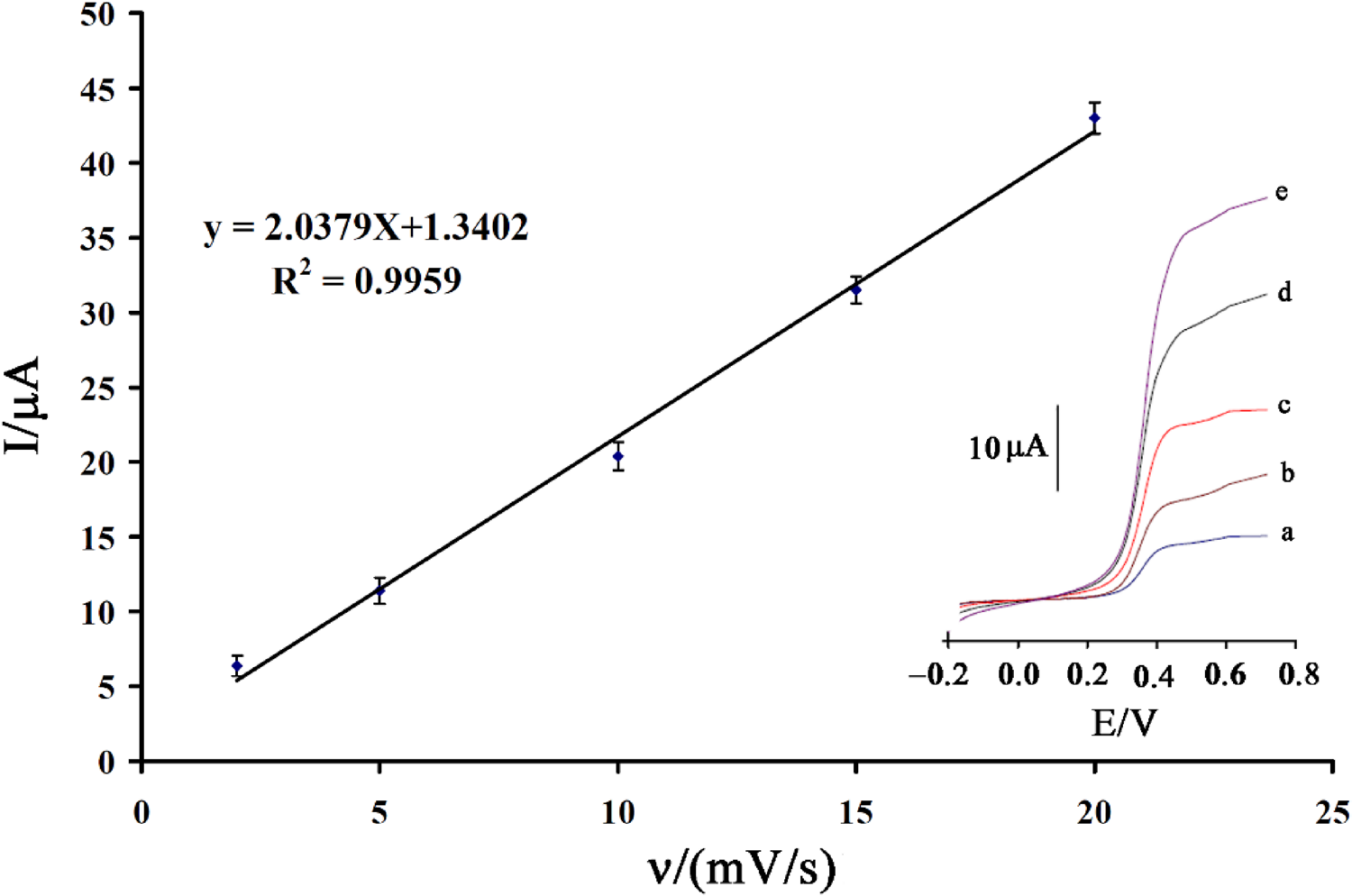
Plot of I_pa_ vs. ν for the oxidation of N-acetylcysteine at the BPOFc/BMPF6/NiO-SWCNTs/CPE (n = 3). Inserts: linear sweep voltammograms of 1.0 mM N-acetylcysteine at various scan rates: (**a**) 2.0, (**b**) 5.0, (**c**) 10.0, (**d**) 15.0 and (**e**) 20 mV/s in 0.1 M PBS (pH 7.0).

The electro-catalytic oxidation signal of 1.0 mM N-acetylcysteine was recorded at the BPOFc/BMPF6/NiO-SWCNTs/CPE surface in a scan rage between 2–20 mV/s (Fig. 4 insert). As shown in Fig. 4, a linear relation between electro-catalytic current of *N*-acetylcysteine at the BPOFc/BMPF6/NiO-SWCNTs/CPE surface with ν, which confirmed an adsorption process for the oxidation of *N*-acetylcysteine at BPOFc/BMPF6/NiO-SWCNTs/CPE surface was observed.

**Figure 5:**
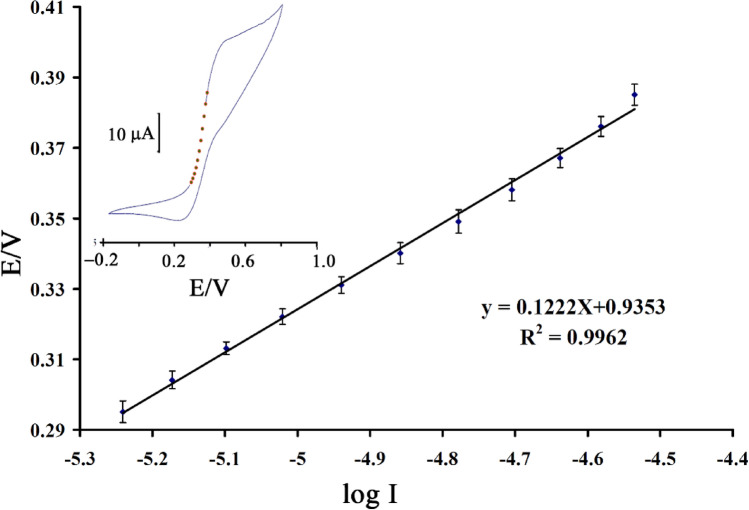
Tafel plots for the BPOFc/BMPF6/NiO-SWCNTs/CPE (pH 7.0) at the scan rate of 20.0 mV/s in the presence of 1.0 mM N-acetylcysteine (n = 3).

A Tafel plot of BPOFc/BMPF6/NiO-SWCNTs/CPE in the presence of 1.0 mM N-acetylcysteine is shown in Fig. 5. The electron transfers coefficient (α) value was measured as ~ 0.516 by the Tafel equation.

The differential pulse voltammetry method was used for the investigation of *N*-acetylcysteine and theophylline in the concentration range of 0.02 to 300.0 μM (sensitivity 0.2079 μA/μM and R^2^ = 0.9961) and 1.0–350.0 μM (sensitivity 0.1643 μA/μM and R2 = 0.9975), respectively. The detection limit (3σ) was set at ~ 8.0 nM and 0.6 μM for *N*-acetylcysteine and theophylline at the surface of BPOFc/BMPF6/NiO-SWCNTs/CPE as a novel electrochemical sensor using (LOD = 3S_b_/m) equation. The BPOFc/BMPF6/NiO-SWCNTs/CPE displayed better dynamic range or the limit of detection for determination of *N*-acetylcysteine compared to another electrochemical methods suggested (Table 1).

**Table 1:** Analytical parameters of suggested sensors for determination of *N*-acetylcysteine.

Electrode	Mediator	LDR (µM)	LOD (µM)	Ref
CPE	N-(3,4-dihydroxyphenethyl)-3,5-dinitrobenzamide + multiwall carbon nanotubes	0.5–200	0.2	^7^
CPE	MgO nanoparticles + acetylferrocene	0.005–50	0.001	^12^
CPE	Ni (II) complex + ZrO_2_ nanoparticle	0.05–600	0.009	^22^
CPE	Copper (II) hexacyanoferrate (III)	120–830	63	^17^
CPE	Carbon nanotube + cobalt salophen complexes	0.1–100	0.05	^18^
CPE	BPOFc/BMPF_6_/NiO-SWCNTs	0.02–300.0	0.008	This work

**Figure 6:**
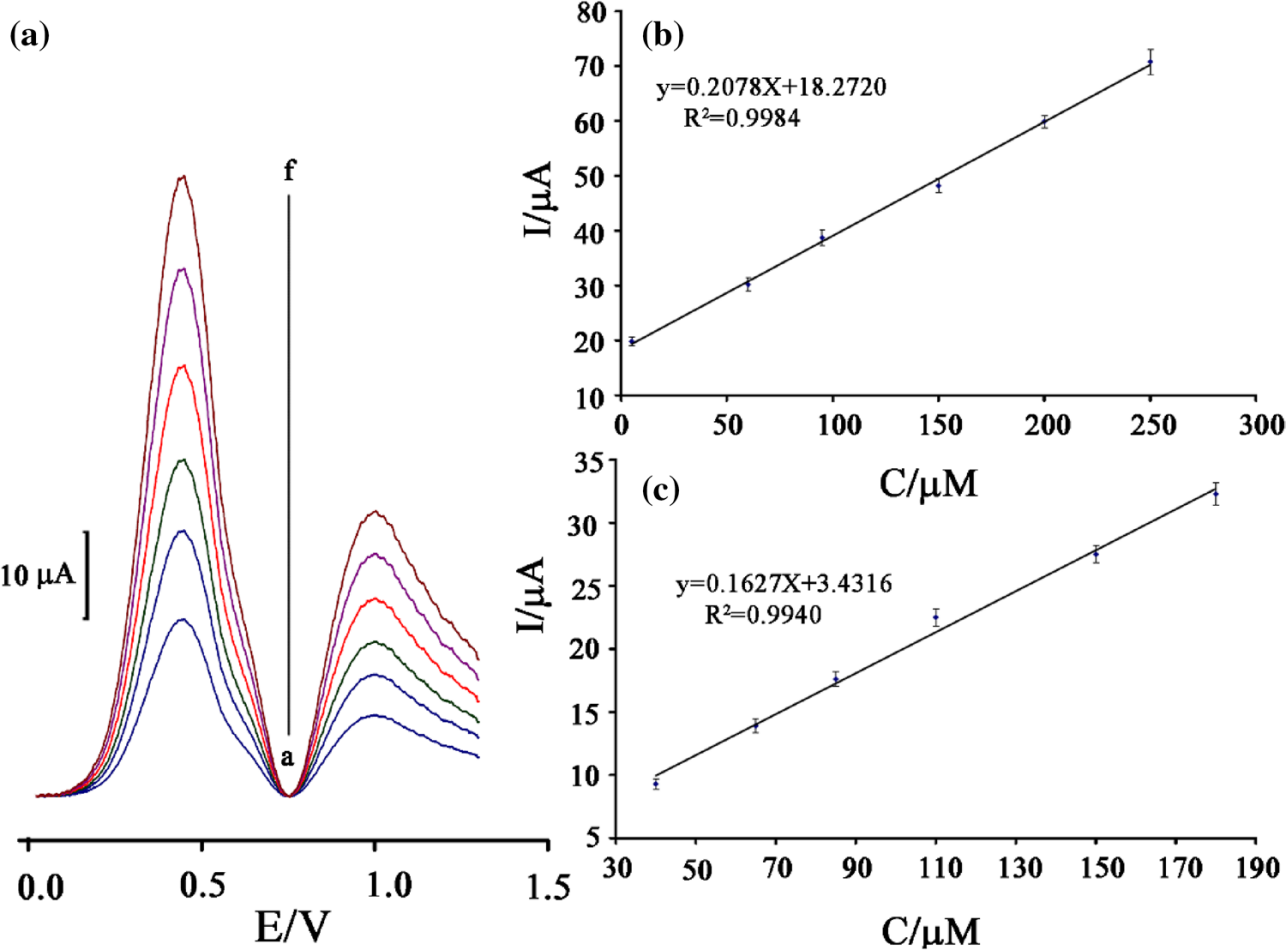
(**a**) differential pulse voltammograms of BPOFc/BMPF6/NiO-SWCNTs/CPE in pH 7.0 containing different concentrations of N-acetylcysteine and theophylline (from inner to outer) mixed solutions of: (a): 5.0 + 40.0 (b): 60.0 + 65.0 + 100; (c): 95.0 + 85; (d): 150.0 + 110.0; (e) 200.0 + 150; and (f) 250 + 180.0 μmol L^−1^ N-acetylcysteine and theophylline, respectively. (**b**) and (**c**) Plots of the electrocatalytic currents as a function of N-acetylcysteine and theophylline concentration, respectively (n = 3).

The differential pulse voltammograms of different concentration of *N*-acetylcysteine and theophylline were measured at the BPOFc/BMPF6/NiO-SWCNTs/CPE surface (Fig. 6A). The voltammograms showed two oxidation peaks separated at potentials of ~ 432 mV and 970 mV that is relative to oxidation of *N*-acetylcysteine and theophylline, respectively. Figure 6B,C showed the plots of oxidation current vs. concentration of drugs. As can be seen, the sensitivity for the simultaneous investigation of *N*-acetylcysteine and theophylline is equal to 0.2078 and 0.1627 μA/μM, which are comparable with sensitivity obtained for the two drugs in linear dynamic range determination. This study revealed that a concurrent determination of *N*-acetylcysteine and theophylline is possible at BPOFc/BMPF6/NiO-SWCNTs/CPE surface with no interference.

The stability of BPOFc/BMPF6/NiO-SWCNTs/CPE was also checked in the presence of 10.0 μM N-acetylcysteine + theophylline solution. BPOFc/BMPF6/NiO-SWCNTs/CPE was stored at the laboratory temperature, and the electro-catalytic signal of BPOFc/BMPF6/NiO-SWCNTs/CPE had no apparent decrease in the first fifteen days. Compared with its first electro-catalytic signal, the response sensitivity remained at 96% after 50 days. The obtained results confirmed good stability of BPOFc/BMPF6/NiO-SWCNTs/CPE as a new electrochemical sensor.

To check the selectivity of BPOFc/BMPF6/NiO-SWCNTs/CPE, the interference effects of some usual biological, cationic, and anionic compounds are investigated in the solution containing 10.0 μM N-acetylcysteine + theophylline. The results indicated that 1000-fold of K^+^, F^-^, Na^+^, Br^−^ and Ca^2+^ and 600-fold of glucose, phenylalanine, and urea have no major influence on the investigation of 20.0 μM N-acetylcysteine.

The ability of the BPOFc/BMPF6/NiO-SWCNTs/CPE was investigated for the study of *N*-acetylcysteine and theophylline in the tablet samples by standard addition technique. The obtained data are shown in Table 2. The recovery data for the analysis of *N*-acetylcysteine and theophylline confirmed good efficacy of BPOFc/BMPF6/NiO-SWCNTs/CPE for the determination of *N*-acetylcysteine as well as theophylline in actual samples.

**Table 2:** Application of BPOFc/BMPF_6_/NiO-SWCNTs/CPE for determination of *N*-acetylcysteine and theophylline in real sample (n = 5).

Sample	Added *N*-acetylcysteine (μM)	Founded *N*-acetylcysteine (μM)	Recovery%	Added theophylline (μM)	Founded theophylline (μM)	Recovery%
Tablet *of N*-acetylcysteine	–	5.11 ± 0.34	–	–	–	–
	15.00	14.89 ± 0.57	99.26	–	–	–
	25.00	24.83 ± 0.67	99.32	–	–	–
Pharmaceutical serum	–	< LOD	–	–	< LOD	–
	5.00	5.17 ± 0.35	103.4	40.00	40.79 ± 0.97	101.97
Tablet of theophylline	–	–	–	20.00	19.75 ± 0.63	98.75
Tap water	–	< LOD	–	–	< LOD	–
	20.00	20.75 ± 0.89	103.75	20.00	19.55 ± 0.94	97.75

### Theoretical studies

#### Electronic properties and structural stability of NiO and SWCNTs

**Figure 7:**
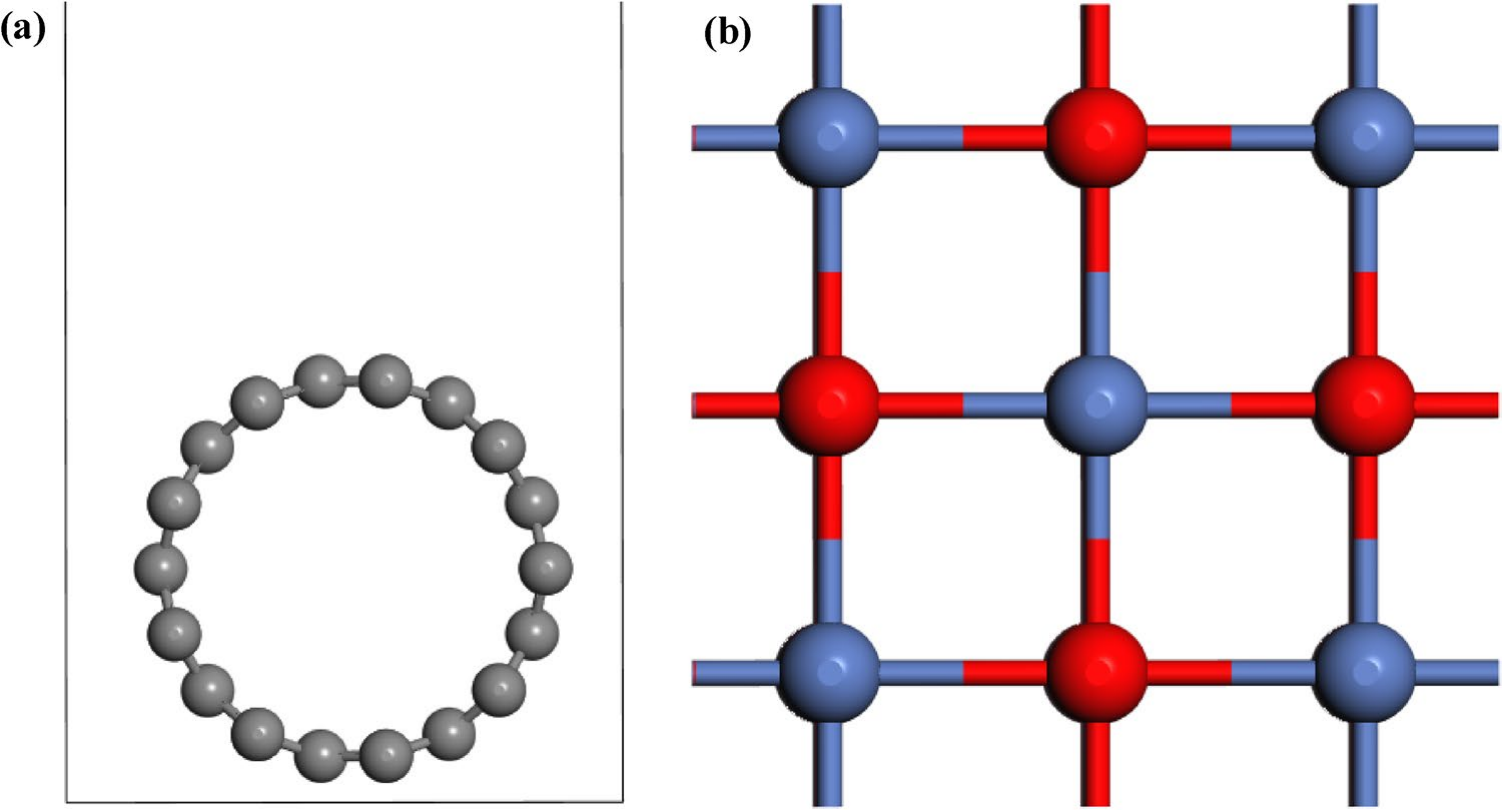
The optimized crystal structures of (**a**) SWCNTs and (**b**) NiO.

Before exploring the adsorption properties of *N-*acetylcysteine using NiO–SWCNTs, the organizational constancy of NiO and SWCNTs was evaluated using Eq. (4) and (5)^61^:

4 $$ E_{form} = \left[ {\frac{1}{x + y}} \right]\left[ {E\left( {NiO} \right) {-} xE\left( {Ni} \right) - yE\left( O \right)} \right] $$

5 $$ E_{form} = \frac{1}{z}\left[ {E\left( {SWCNTs} \right) {-} zE\left( C \right)} \right] $$

where $E\left( {NiO} \right)$, $E\left( {SWCNTs} \right)$, $E\left( {Ni} \right)$, $E\left( O \right)$, and $E\left( C \right)$ are the total energies of NiO, SWCNTs, isolated Ni, O, and C atoms, respectively. Moreover, x, y, and z are the number of Ni, O and C atoms, respectively. The formation energy of NiO and SWCNTs was calculated as − 9.83 and − 8.71 eV, respectively, confirming their stable structure. The stoichiometry of C, Ni and O in NiO–SWCNTs was 32.55, 53.00 and 14.25%. The SWCNTs used in this study contained 72 carbon atoms (Fig. 7).

**Figure 8:**
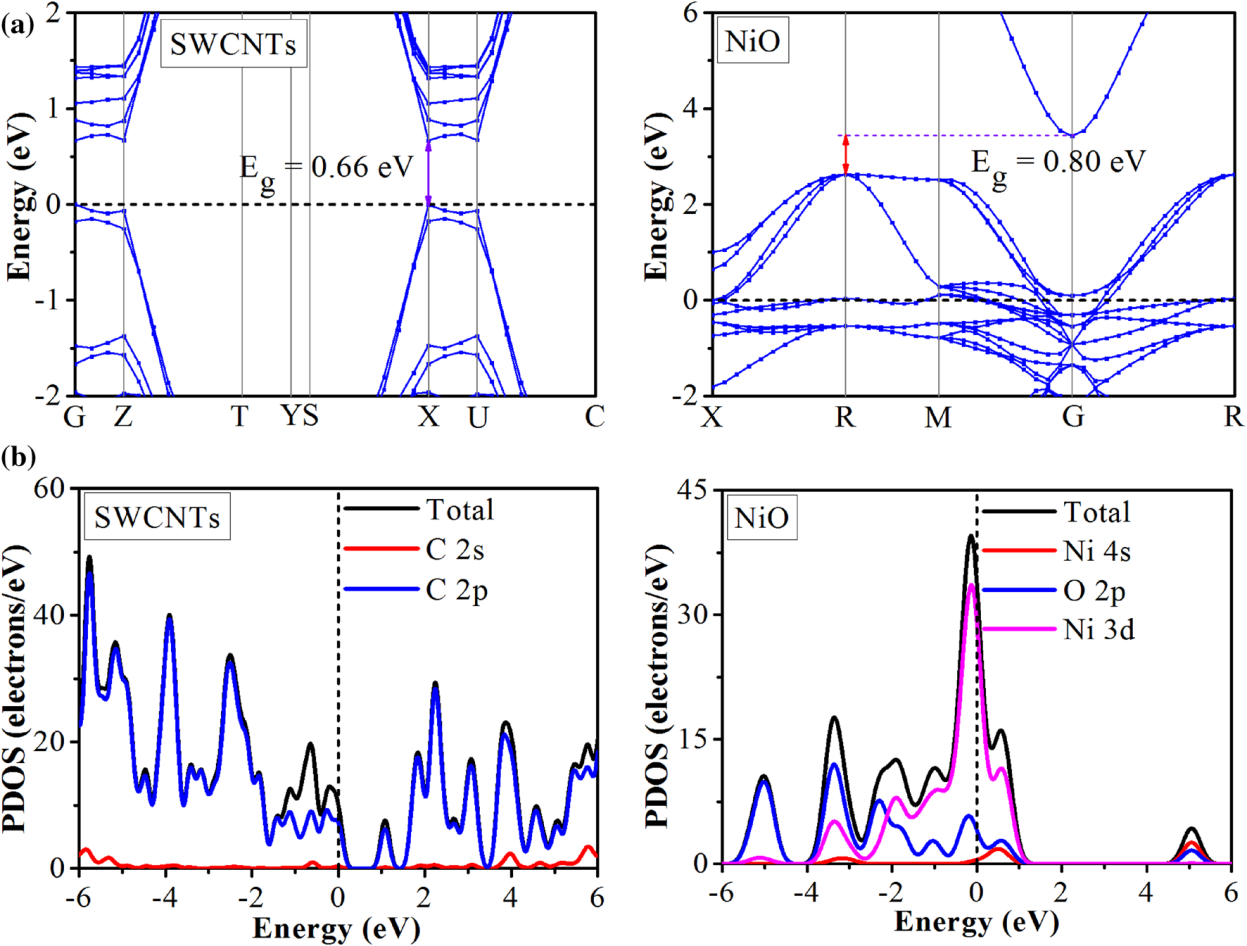
(**a**) Electronic band structures and (**b**) projected density of states (PDOS) of SWCNTs and NiO. The Fermi energy level was set to 0.0 eV as a black dashed line.

Several types of C–C bonds were observed in the SWCNTs with different bond lengths of 1.40–2.44 Å (bonds shared between two hexagons) and 1.44 Å (the bond shared between a hexagon and pentagon), which were comparable with other studies^62^. The Ni–O bond length of 2.10 Å was in agreement with the earlier results (2.08 Å)^63^. The electronic properties of NiO and SWCNTs were described based on the HLG. The band structure of NiO in Fig. 8 revealed that the SWCNT is a semiconductor with an *E*_*g*_ of 0.67 eV. The LUMO and HOMO of the SWCNT were − 5.01 and − 5.81 eV, respectively.

From the DOS plot of NiO, an HLG of 0.80 eV was revealed. The obtained PDOS results showed that the 3d orbitals of the surface Ni were mainly located at the HOMO, while at the LUMO, the hybridization was mostly contributed by Ni 4 s orbitals. The PDOS results suggested that the Ni 3d orbitals play a key influence on the adsorption process.

**Figure 9:**
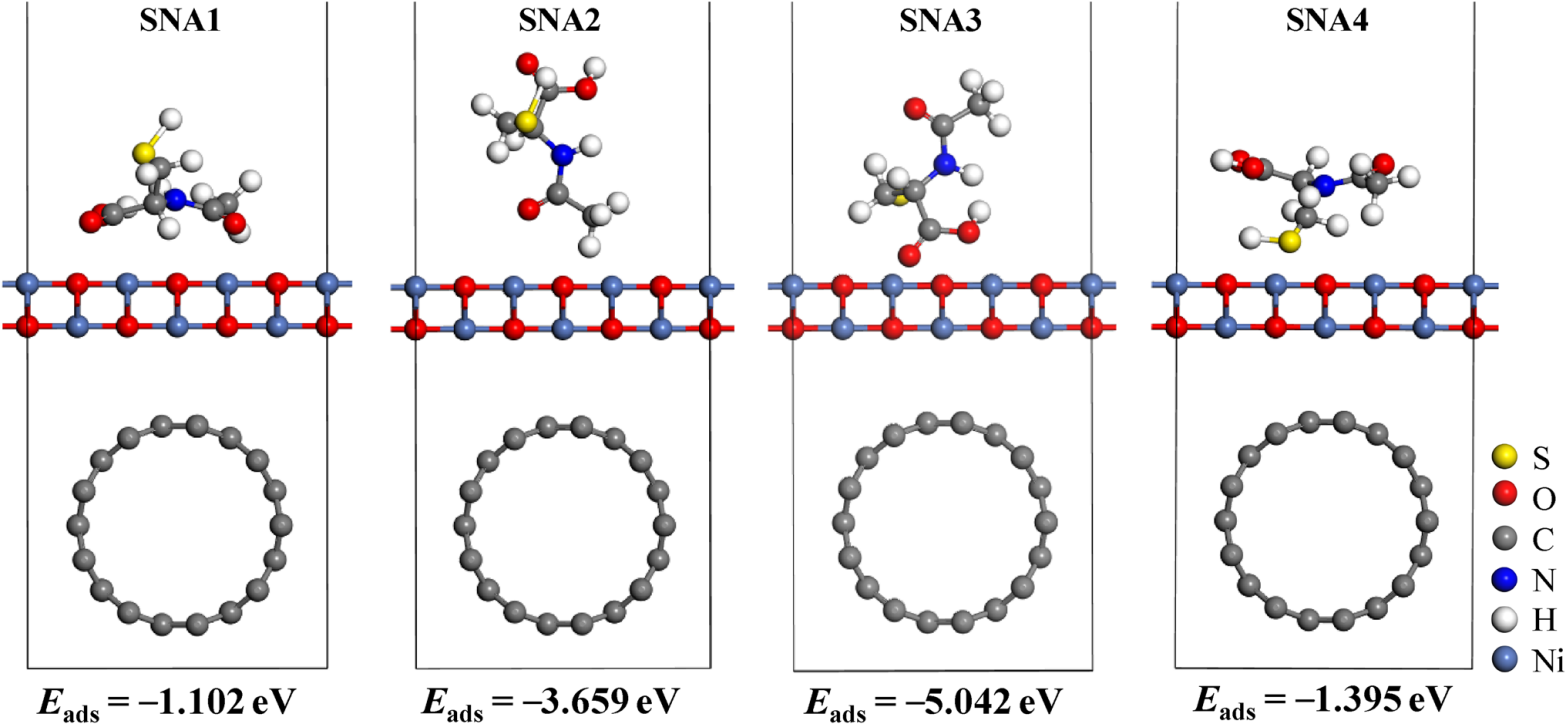
Various relaxed adsorption configurations of *N-*acetylcysteine onto NiO–SWCNTs surface.

Several configurations were explored to find the most feasible adsorption sites where four local minima were obtained (Fig. 9).

Based on the *E*_ads_ calculations, the four configurations of *N-*acetylcysteine adsorption onto NiO–SWCNTs were exothermic processes with negative adsorption energies ranging between − 1.102 and − 5.042 eV (Table 3). Moreover, the adsorption energy varies owing to the interactions of *N-*acetylcysteine molecule with several adsorption sites with the NiO–SWCNTs. As presented in Table 3, the four relaxed configurations with more negative adsorption energy values and small interaction distances (ranging from 1.689 to 1.980 Å) between the *N-*acetylcysteine and NiO–SWCNTs, signify strong interactions and stability. This strong interaction indicates that the NiO–SWCNTs is a prominent sensor for the adsorption of *N-*acetylcysteine with good response to all the adsorption sites considered. Moreover, the more negative adsorption energy value suggests that the reaction will release more energy. Among these configurations, the most stable (SNA3) is where the acidic end is bonded strongly with the interfacial Ni atoms of the substrate^64–66^.

**Table 3:** The adsorption energy (*E*_ads_), adsorption distance (*D*), LUMO energy $\left( {E_{LUMO} } \right)$, HOMO energy $\left( {E_{HOMO} } \right)$, HOMO–LUMO gap (HLG), change of HLG (|ΔHLG|), charge transfer (*QT*) and conductivity $\left( \sigma \right)$ for the adsorption of *N-*acetylcysteine molecule onto NiO–SWCNTs surface.

	SNA1	SNA2	SNA3	SNA4
*E*_ads_ (eV)	− 1.102	− 3.659	− 5.042	− 1.395
*D* (Å)	1.980	1.689	1.512	1.816
$E_{HOMO}$(eV)	− 3.91	− 3.53	− 3.25	− 3.69
$E_{LUMO}$(eV)	− 3.40	− 3.17	− 3.01	− 3.25
HLG (eV)	0.51	0.36	0.24	0.44
|ΔHLG| (%)	63.09	68.33	69.83	65.84
*QT* |e|	1.11	1.18	1.21	1.14
$\sigma$	5.20 × 10^−5^	9.47 × 10^−4^	9.64 × 10^−3^	2.01 × 10^−4^

**Figure 10:**
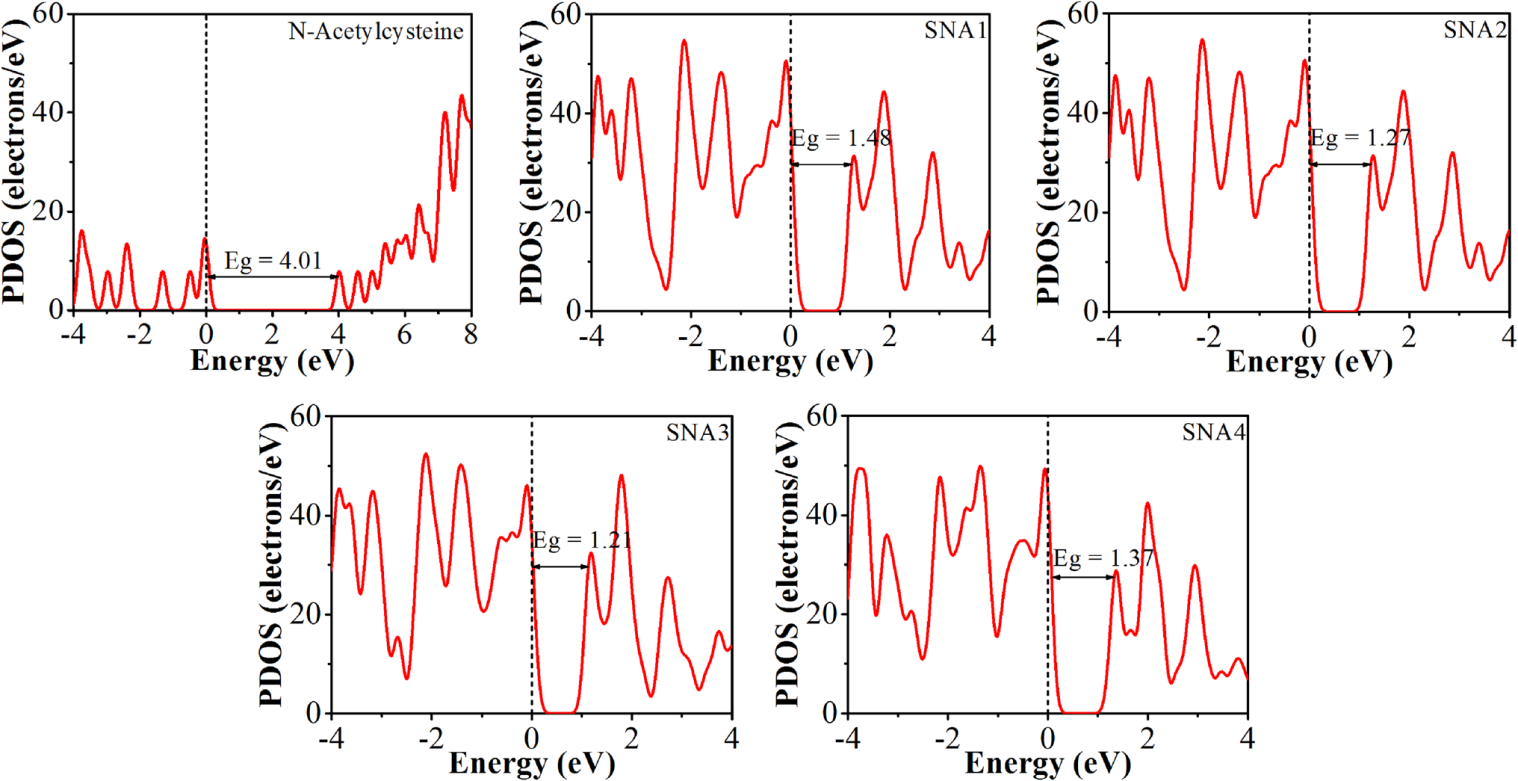
DOS of *N-*acetylcysteine molecule before and after adsorption on the NiO–SWCNTs surface. The Fermi energy level is set to zero as reference energy and shown as a dashed black line.

The interaction between the *N-*acetylcysteine molecule and NiO–SWCNTs was anticipated to alter the electronic property of *N-*acetylcysteine, which could be understood by the variation in its energy band gap^67–69^. The electronic property of *N-*acetylcysteine molecule was studied based on the HLG and density of states (DOS) spectrum (Fig. 10). The DOS of *N-*acetylcysteine molecule possesses a broad HOMO and LUMO separated by a wide HLG (Fig. 10). After adsorption, the *N-*acetylcysteine molecule introduced sharp occupied bands in the HLG of all the configurations. The TDOS results revealed similar changes, which indicated that NiO–SWCNTs might be an effective sensor towards the *N-*acetylcysteine molecule. The adsorption of *N-*acetylcysteine molecule shifted the HOMO levels to a higher energy, whereas the LUMO levels remained unaffected. Thus, the HLG value of *N-*acetylcysteine molecule was significantly reduced compared to its isolated molecule. The average HLG variation (|ΔHLG| (%)) upon adsorption of *N-*acetylcysteine molecule onto NiO–SWCNTs surface is connected with the sensitivity of adsorbent, as well as modifying its electrical conductivity. The |ΔHLG| (%) of 63.09, 68.33, 69.83, and 65.84% for configurations SNA1, SNA2, SNA3, and SNA4, respectively (see Table 3), signified high sensitivity of NiO–SWCNTs towards the adsorption of *N-*acetylcysteine molecule on its surface. From the HLG variation result, it was established that the sensing response of NiO–SWCNTs towards *N-*acetylcysteine molecule was observed to be rather higher for SNA3 configuration. Further understanding into the bonding mechanisms between the *N-*acetylcysteine molecule and NiO–SWCNTs was obtained by analyzing the DOS of *N-*acetylcysteine molecule before and after adsorption onto the NiO–SWCNTs surface (Fig. 10). After adsorption, the DOS of *N-*acetylcysteine molecule was broadened owing to the strong hybridization with the adsorbed Ni ion. This showed a chemisorption state of *N-*acetylcysteine molecule.

The migration of charge carriers between *N-*acetylcysteine molecule and NiO–SWCNTs induces a variation in HOMO–LUMO gap energy. Also, the stability and electrical conductivity of sensors are exponentially related to the HOMO–LUMO gap energy^70^. For that reason, the *N-*acetylcysteine molecule can be sensed by evaluating the change in the conductivity of the.

*N-*acetylcysteine molecule before and after adsorption^71^:

6 $$ \sigma \alpha exp \left( {\frac{{{-}E_{g} }}{2kT}} \right) $$

where $\sigma$, *k,*$E_{g}$ and *T* are the electrical conductivity, Boltzmann’s constant, bandgap energy and thermodynamic temperature, respectively. According to this equation, smaller HLG values lead to a larger electrical conductivity. The electrical conductivity before adsorption was 2.86 × 10^–9^. Therefore, the electrical conductivity of SNA1, SNA2, SNA3 and SNA4 configurations was higher after adsorption.

**Figure 11:**
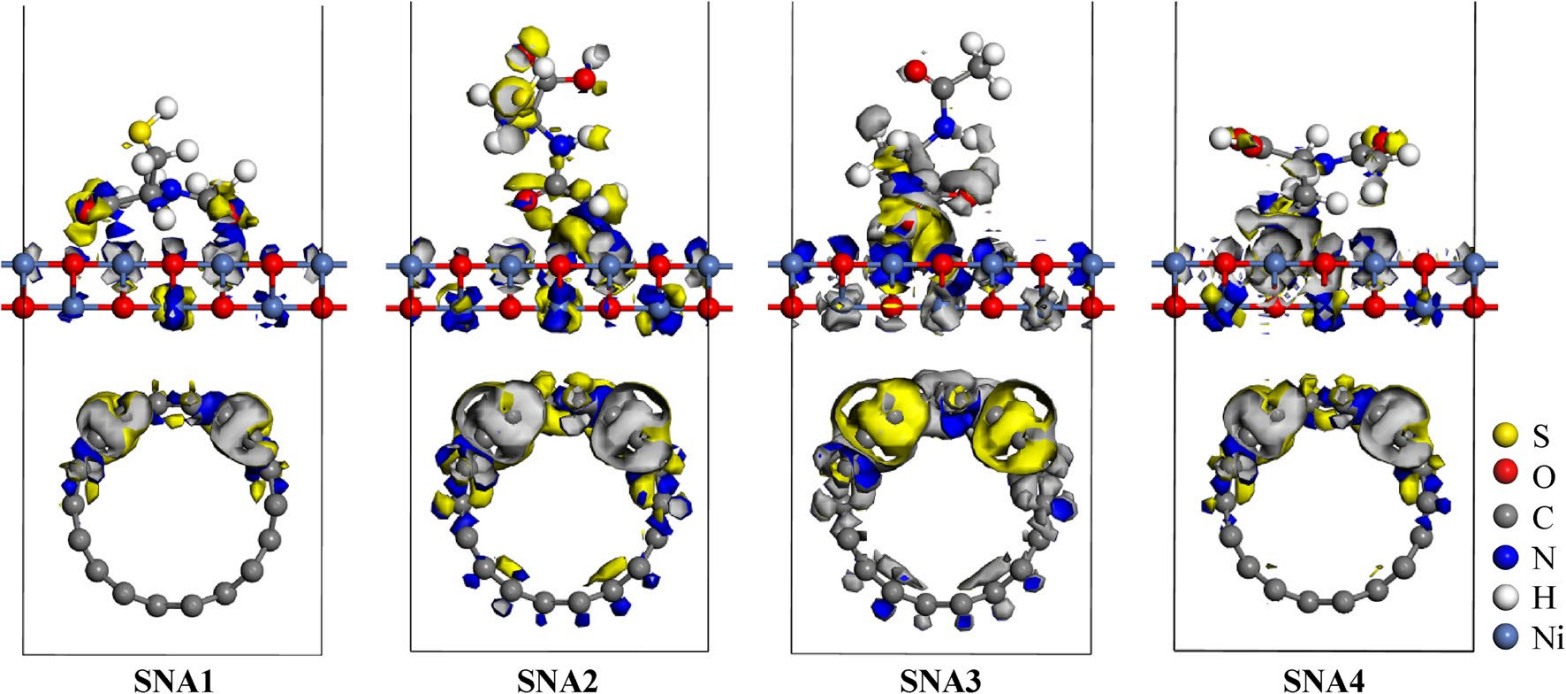
3D charge density differences upon N-acetylcysteine molecule adsorption on the NiO–SWCNTs surface showing regions of charge depletion (yellow) and accumulation (blue) with an isovalue of 0.007 e Å^−3^.

To evaluate the interactions between the *N-*acetylcysteine and NiO–SWCNTs, the three-dimensional (3D) charge density difference was calculated, as given in Fig. 11.

In this 3D charge density difference plot, the electron enrichment and depletion are shown as blue and yellow isosurfaces, respectively. The electronic interaction largely occurred at the top of Ni atoms of the NiO–SWCNTs nanocomposite, which was in direct contact with the *N-*acetylcysteine molecule. The electrons transfering from the *N-*acetylcysteine molecule to NiO–SWCNTs indicated that the Ni atoms were oxidized during the adsorption process. Since the electron accumulation sites were mostly located at the interface, they confirmed that the bond between *N-*acetylcysteine molecule and NiO–SWCNTs was of a covalent nature. However, less electron density was observed at the NiO–SWCNTs interface, signifying that the SWCNTs was less influenced electronically by the interaction with NiO nanoparticle. The interactions between the *N-*acetylcysteine molecule and NiO–SWCNTs indicates a substantial charge transfer, which was evaluated grounded on the Hirshfeld charge analysis. The charge migration analysis of SNA1, SNA2, SNA3, and SNA4 configurations was found to be 1.11, 1.18, 1.21 and 1.14 |e|, respectively. A positive value of Hirshfeld charge analysis was observed for the four interaction sites considered in this study. This further confirmed electrons transfer from the *N-*acetylcysteine molecule to NiO–SWCNTs.

**Figure 12:**
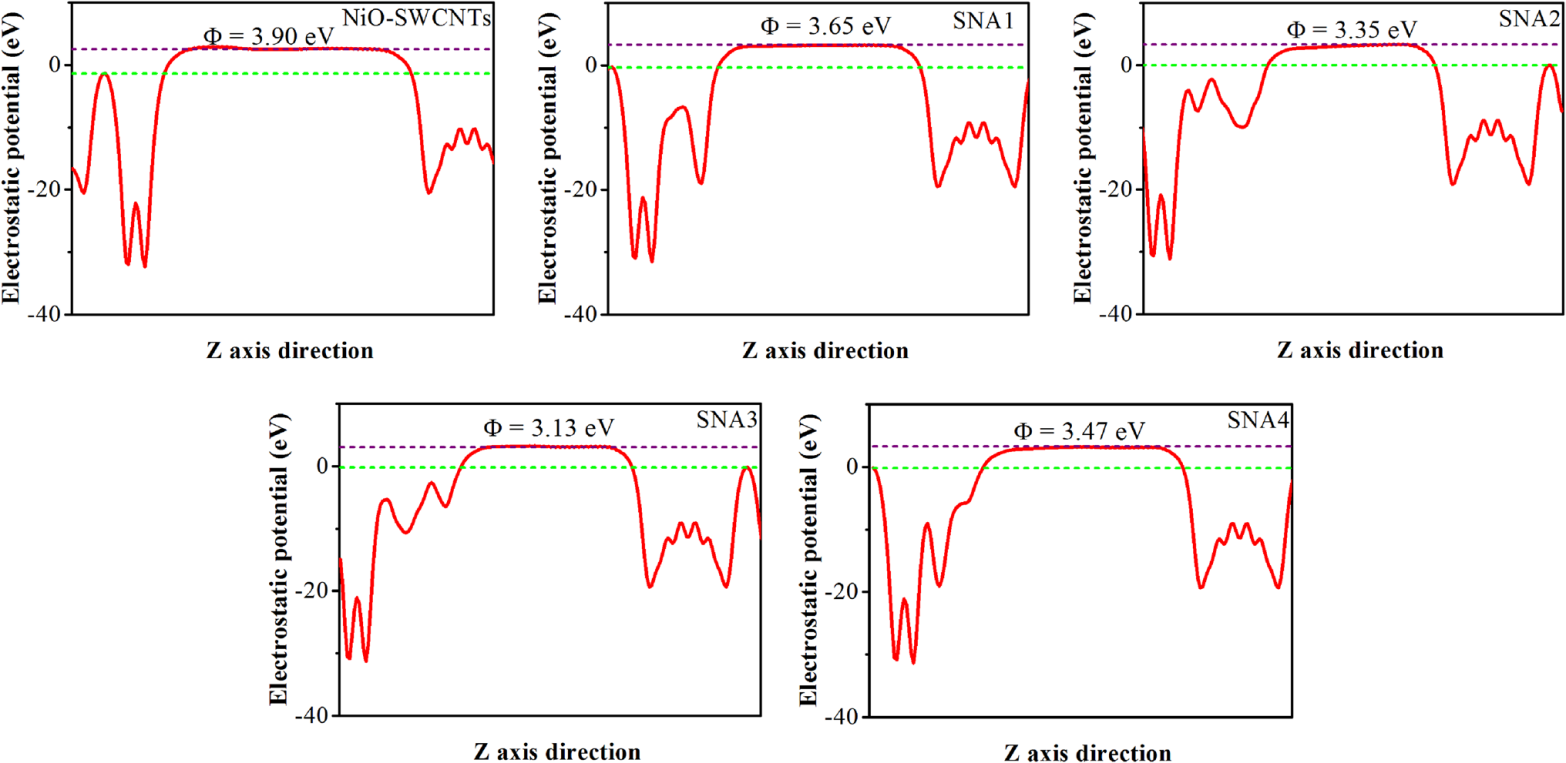
The calculated work function ($\Phi$) of *N*-acetylcysteine molecule adsorption on the NiO–SWCNTs surface.

The changes of work function connected to the charge transfer between *N-*acetylcysteine molecule and NiO–SWCNTs was used to evaluate the sensitivity of NiO–SWCNTs towards the adsorption of *N-*acetylcysteine molecule. The field emission property was altered due to the work function change of NiO–SWCNTs before and after the adsorption of *N-*acetylcysteine. According to Fig. 12, the work function of NiO–SWCNTs was decreased after *N-*acetylcysteine molecule adsorption due to the charge migration from the *N-*acetylcysteine molecule to the NiO–SWCNTs surface.

## Conclusion

The electro-catalytic interaction between BPOFc and *N*-acetylcysteine was studied at the BPOFc/BMPF6/NiO-SWCNTs/CPE surface. The cyclic voltammograms data confirms the good selectivity and high sensitivity of BPOFc/BMPF6/NiO-SWCNTs/CPE for determination of *N*-acetylcysteine. Moreover, the most prominent adsorption site, sensitivity, conductivity, charge transfer, electronic and structural properties of *N-*acetylcysteine molecule adsorption onto NiO–SWCNTs surface was studied using DFT studies. The negative adsorption energies in the range of –1.102 to –5.042 eV and suitable charge transfer confirmed the stability of *N-*acetylcysteine adsorption at NiO–SWCNTs surface. In addition, the adsorption of *N-*acetylcysteine molecule was chemisorption. Therefore, the most prominent adsorption site of *N-*acetylcysteine molecule at NiO–SWCNTs surface was when the acidic end of *N-*acetylcysteine molecule was adsorbed at NiO–SWCNTs surface. The theoretical investigation established the high electrical conductivity of NiO-SWCNTs and suggested this nano-composite as a conductive binder for modification of carbon paste electrode. The BPOFc/BMPF6/NiO-SWCNTs/CPE can be detected as *N*-acetylcysteine in the presence of theophylline with limits of detection 8.0 nM and 0.5 μM. The finding of this study offers useful information to design novel NiO–SWCNTs-based sensors for sensing toxic molecule.
