# Author Correction: Electro-catalytic amplified sensor for determination of N-acetylcysteine in the presence of theophylline confirmed by experimental coupled theoretical investigation

**DOI:** 10.1038/s41598-021-97725-2

**Published:** 2021-09-06

**Authors:** Mohsen Keyvanfard, Hassan Karimi‑Maleh, Fatemeh Karimi, Francis Opoku, Ephraim Muriithi Kiarii, Poomani Penny Govender, Mehdi Taghavi, Li Fu, Aysenur Aygun, Fatih Sen

**Affiliations:** 1 Department of Chemistry, Majlesi Branch, Islamic Azad University, Majlesi, Iran; 2 School of Resources and Environment, University of Electronic Science and Technology of China, Xiyuan Ave, Chengdu, 611731 People’s Republic of China; 3 Department of Chemical Engineering, Quchan University of Technology, Quchan, Iran; 4 Department of Chemistry, University of Johannesburg, Doornfontein Campus, P.O. Box 17011, Johannesburg, 2028 South Africa; 5 Polymer Chemistry Research Laboratory, Faculty of Science, Shahid Chamran University, 61357‑43337 Ahvaz, Iran; 6 College of Materials and Environmental Engineering, Hangzhou Dianzi University, Hangzhou, 310018 China; 7 Sen Research Group, Biochemistry Department, Faculty of Arts and Science, Dumlupınar University, Evliya Çelebi Campus, 43100 Kütahya, Turkey

Correction to: *Scientific Reports* 10.1038/s41598-020-79872-0, published online 13 January 2021

In the original version of this Article, Francis Opoku, Ephraim Muriithi Kiarii and Poomani Penny Govender were incorrectly affiliated with ‘Department of Chemical Engineering, Quchan University of Technology, Quchan, Iran’. The correct affiliation for these authors is listed below.

Department of Chemistry, University of Johannesburg, P.O. Box 17011, Doornfontein Campus, Johannesburg, 2028, South Africa

The original Article has been corrected.

